# The effect of baicalein on Wnt/β-catenin pathway and miR-25 expression in Saos-2 osteosarcoma cell line

**DOI:** 10.3906/sag-2001-161

**Published:** 2020-06-23

**Authors:** Esra ÖRENLİLİ YAYLAGÜL, Celal ÜLGER

**Affiliations:** 1 Department of Nutrition and Dietetics, Faculty of Health Sciences, Aydın Adnan Menderes University, Aydın Turkey; 2 Department of Biology, Faculty of Arts and Science, Aydın Adnan Menderes University, Aydın Turkey

**Keywords:** MicroRNA, miR-25, baicalein, osteosarcoma, Wnt/β-catenin pathway, gene expression

## Abstract

**Background/aim:**

Osteosarcoma is the most common primary bone malignancy that occurs frequently in children and adolescents. Baicalein, a flavonoid that has attracted great attention in recent years with its strong antitumor activity, shows a wide range of biological and pharmaceutical effects.MicroRNAs have been found to be involved in many critical processes in cancers. This study aimed to investigate the effect of baicalein and miR-25 on Wnt/β-catenin signaling pathway of osteosarcoma cell line Saos-2.

**Materials and methods:**

Cell viability was assessed, and qRT-PCR and Western blot were performed to study the effects of baicalein on expression of Wnt/β-catenin signaling pathway-realted genes (β-catenin, GSK-3β, and Axin2) of Saos-2 cells.

**Results:**

Our results indicated that baicalein can inhibit the proliferation (IC50 value 35 µM), regulate Wnt/β-catenin pathway and also increase miR-25 expression of Saos-2. Baicalein and also miR-25 decreased the expression of β-catenin and Axin2, while increasing the expression of GSK-3β. Down regulation of miR-25 decreased the expression of GSK-3β, while β-catenin and Axin2 expression increased.

**Conclusion:**

These findings demonstrate that baicalein may target genes related to the Wnt/β-catenin pathway by regulating miR-25 expression and may be a potential Wnt/β-catenin pathway inhibitor for osteosarcoma therapy.

## 1. Introduction

Osteosarcoma (OS) is the widespread primary bone malignancy that occurs generally in children and adolescents [1], but can also occur at any age. The effective treatment of some natural chemicals used traditionally for human diseases is striking. Researchers have focused on the ability of natural components to regulate signal pathways [2]. *Scutellaria baicalensis* is a widely used plant because of its strong anticancer effects. The main components of Scutellaria are bioactive flavones which are baicalein, baicalin, and wogonin. These phytochemicals have been demonstrated by studies that suppress tumor growth [3,4]. Baicalein is a flavonoid that is extracted from the roots of *Scutellaria baicalensis *[5]. It shows a wide variety of biological and pharmaceutical effects and has accumulated interest in recent years due to its potent antitumor activity. Baicalein has been shown to exert antitumor effects by suppressing metastasis [6] and inducing apoptosis [5, 7] in many types of cancer.

MicroRNAs (miRNAs, miRs) are 18-25-nucleotide length single-stranded noncoding RNAs that are critical for various biological processes [8, 9]. The microRNA nucleotide sequences can partially or completely bind to the 3ʹ-untranslated regions (3ʹ-UTR) of the targeted mRNAs to suppress gene expression [10]. Because of these properties, a single microRNA has many target genes. Generally, many of these targets are associated with various signal pathways such as Notch, MAPK, PI3K/Akt, Wnt and Jun/FOS in osteosarcoma [9] and every stage of proliferation, differentiation, and apoptosis of cells [7,11]. Irregularities in the expression of miRNAs are associated with cancers [12], autoimmune disorders [13], developmental and neurological disorders [14]. 

MicroRNA-25 (miR-25) is one of the microRNAs associated with several human diseases such as cancer [15,16]. It is involved in the development and the progression of cancers [17]. The expression of miR-25 is low in colon cancer tissues and inhibits cell proliferation and migration. It means miR-25 may function as a tumor suppressor in colon cancer [18]. Studies have shown that microRNA-25 has high expression in various human malignant solid tumors such as stomach [19], prostate [20] and liver [21]. In such cancers, miR-25 acts as an oncogene and poses potential proliferative, antiapoptotic and cell cycle stimuli. The effects of miR-25 on cancer are complex because it may therefore function as an oncogenic or tumor suppressor in different types of cancer.

Wnt signaling pathways play an important role in the regulation of different processes such as cell proliferation, migration, determination of cell flux, and self-renewal of stem cells [22]. Abnormal activation of Wnt signaling pathways leads to the emergence of many cancers [23]. There are studies supporting miRNAs that regulate Wnt signaling components are associated with various cancers [24]. It has been noted that miR-25 plays an important role in the Wnt signaling pathway [17,25]. Oncogenesis due to abnormal miRNA expression can occur through resulting overexpression of oncogenic miRNAs or genetic loss of tumor suppressor miRNAs [24]. Studies have shown that Wnt/β-catenin signaling pathway activation is common in OS cells and that abnormal activation plays an important role in tumorigenesis and chemotherapeutic responses [26]. 

There is no study showing the effect of baicalein on miR-25 in osteosarcoma. This study aimed to investigate the effect of baicalein and miR-25 on Wnt/β-catenin signaling pathway of osteosarcoma cell line Saos-2. In this study, we report the effects of baicalein on cell proliferation in the osteosarcoma cell line Saos-2, as well as on the expression of miR-25 and Wnt/β-catenin signal pathway-related genes, all of which were investigated. This study shows that baicalein acts on miR-25 to regulate the Wnt/β-catenin signalling pathway. Besides, we present that there is a correlation between miR-25 expression and Wnt/β-catenin signaling pathway-related genes.

## 2. Materials and methods

The study designed to determine the effect of Baicalein on miR-25 expression, as well as the effect of baicalein and miR-25 on the Wnt/β-catenin signaling pathway-related genes in osteosarcoma cell line Saos-2.

### 2.1. Cell culture

The Saos-2 cell line (Saos-2/An1, 02111901) was obtained from ŞAP Institute (Republic of Turkey Ministry of Food Agriculture and Livestock, Ankara, Turkey). Cells were plated in 25 cm2 or 10 cm2 tissue culture dishes in Mc Coy’s 5A medium (supplemented with 15% FBS, 1% pen/strep). Cells were grown at 37 °C under humidified 5% CO2–95% air at one atmosphere. Cells were allowed to grow until 80% confluence.

### 2.2. Cell viability assay

Cell viability was quantified using the Muse™ Cell Analyzer with the Muse™ Count and Viability Kit (Merck-Millipore, Burlington, MA, USA) according to the manufacturer. Saos-2 cells were plated onto 12-well plates (2 × 105 cells/well) and allowed to grow for 24 h. Then, cells were severally treated with baicalein concentrations (0, 4, 10, 15, 20, 35, 50 and 80 μM) for 48 h. Treated cells with baicalein were suspended in PBS. Then, a working solution was added to the cell suspension and incubated for 5 min at room temperature in the dark. Cell viability was determined as percent viability compared with the control samples.

### 2.3. Cell transfection assay

Lipofectamine 2000 (Invitrogen) was used to perform cell transfection. Before transfection, Saos-2 cells were cultured in Mc Coy’s 5A medium overnight. For miR-25 expression analysis, Saos-2 cells were transfected with miR-25 mimic (5 nM) and mimic negative control or miR-25 inhibitor (30 nM) and inhibitor negative control according to the manufacturer’s protocol. MiR-25 mimic group cells were transfected with synthetic hsa-miR-25 (mirVana™ miRNA mimic); mimic control group transfected with hsa-miR-25 mimic negative control (mirVana™ miRNA mimic negative control #1); inhibitor group transfected with synthetic anti-miR-25 (mirVana™ miRNA inhibitor); inhibitor control group transfected with anti-miR-25 negative control (mirVana™ miRNA negative control). Then, 250 μl of miR/lipofectamine compound was added to Opti-MEM culture media and incubated for 8 h. After incubation, the transfection medium (Opti-MEM) for anti-miR-25 was replaced by fresh Mc Coy’s 5A medium supplemented with or without baicalein (35 µM) to see baicalein’s effect. Also the transfection media for miR-25 mimic, miR-25 mimic negative control and anti-miR-25 negative control was replaced by fresh Mc Coy’s 5A medium supplemented without baicalein. Following this, Saos-2 cells were cultured for 48 h prior to the subsequent assays. Cells were collected and lysed in order to extract total RNA and protein for other analyses.

### 2.4. Quantitative real-time PCR (qRT-PCR)

Total RNA was extracted using the TRIzol® Reagent (Thermo Fisher Scientific Inc., Waltham, MA, USA) according to the manufacturer’s instructions. cDNA was reverse-transcribed using the TaqMan® Reverse Transcription Reagents (Applied Biosystems, Waltham, MA, USA) and was amplified by SYBR® Green PCR Master Mix (Applied Biosystems, Waltham, MA, USA). qRT-PCR was prepared following the instructions. Each sample was analysed using a StepOne™Real-Time PCR System (Thermo Fisher Scientific Inc., Waltham, MA, USA). Gene copy number data was analysed in accordance with the 2-ΔΔCt method [27] relative to the expression of GAPDH and compared with the control. Primers sequences for specific genes were as follows: 

*β-catenin *forward 5’-TTCTGGTGCCACTACCACAGC-3’, reverse 5’-TGCATGCCCTCATCTAATGTC-3’; *Axin2* forward 5’-GAATGAAGAAGAGGAGTG-3’, reverse 5’-AAGACATAGCCAGAACC-3’; *GAPDH* forward 5’-TGCACCACCAACTGCTTAGC-3’, reverse 5’-GGCATGGACTGTGGTCATGAG-3’.

### 2.5. MicroRNA quantification by qRT-PCR

MiR-25 expression levels quantification using the TaqMan microRNA assays was performed to the manufacturer’s instructions. cDNA was reverse transcribed from total RNA using specific miR-25 primers (Applied Biosystems, Waltham, MA, USA). PCR products were amplified from cDNA samples using the TaqMan MicroRNA Assays and Universal PCR Master Mix II (Applied Biosystems, Waltham, MA, USA). The real-time PCR results were normalized against an endogenous control *RNU6B* (Applied Biosystems, Waltham, MA, USA). 

### 2.6. Western blot assay

Saos-2 cells were separately treated baicalein (35 μM) with or without miR-25 inhibitor (30 nM), miR-25 mimic (5 nM) and their negative controls for 48 h. Total of 2 × 106 (for baicalein treatment) or 2 × 105 (for transfection) cells of each group were suspended in ice-cold lysis and centrifuged at 10000 g at 4oC for 20 min. Protein solutions from cells were collected, and protein concentration in the resulting lysate was determined using the Bradford assay. Each sample was separated by SDS-PAGE and transferred onto the polyvinylidene fluoride membranes (Millipore, USA). The membranes were subsequently blocked in skim milk [5% in Tris-buffer with Tween® 20; (TBST buffer)] at 25°C for 1 h. Then membranes incubated at 4 °C overnight with antibodies against β-catenin, Axin2, GSK-3β, GAPDH (Cell Signaling Technology, Danvers, MA, USA) or Actin (Santa Cruz Biotechnology, Inc., Dallas, TX, USA) in TBST containing 5% defatted milk separately. The membranes were then incubated with proper antirabbit IgG AP-linked secondary antibody (Cell Signaling Technology, Danvers, MA, USA) for 1 h at room temperature. Finally, the bands were detected with BCIP/NBT Alkaline Phosphates Colour Development Kit (Thermo Fisher Scientific Inc., Waltham, MA, USA) and analyzed for optical density using NIH ImageJ software.

### 2.7. Statistical analysis

Statistical analyses were performed using the SPSS 20.0 software (IBM Corp., Armonk, NY, USA). Comparisons between the 2 groups were analyzed using the Student’s t-test. Statistical significance was considered as P < 0.05.

## 3. Results

### 3.1. The effect of baicalein on Saos-2 cell proliferation

**Figure 1:**
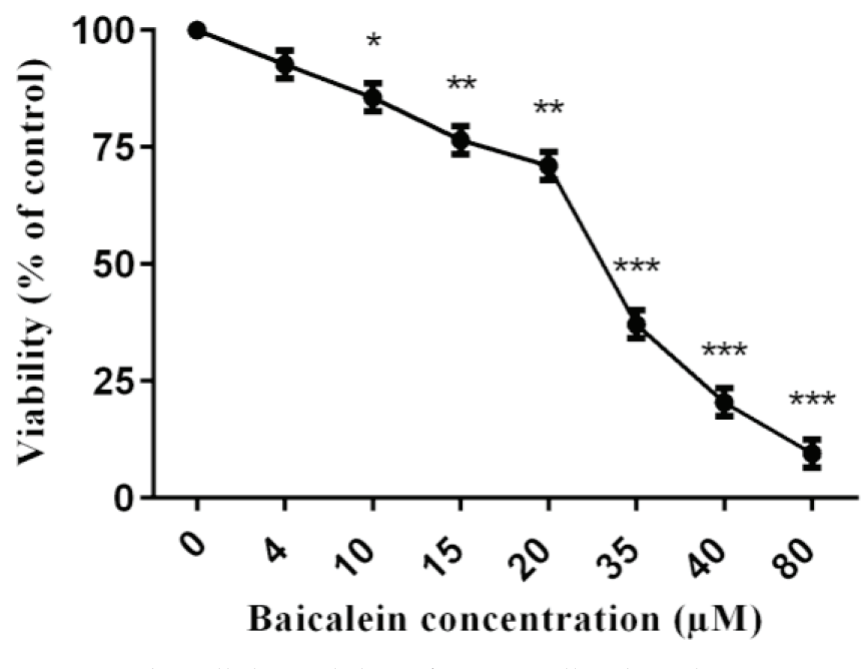
The cellular viability of Saos-2 cells. The values represent a mean ± standard deviation of 3 independent experiments performed in triplicate (*P < 0.05 **P < 0.01 and ***P < 0.001).

The proliferation of Saos-2 osteosarcoma cells treated with different concentrations of baicalein was determined by Muse™ Count & Viability Assay. After 48 h of treatment with baicalein, the proliferation of Saos-2 cells was compared with the untreated group (control). We found a statistically significant decrease in the proliferation of cells depending on concentrations (P < 0.05) (Figure 1). When we look at the viability graph (Figure 1), we can see an obvious decrease over 20 μM baicalein concentration. The IC50 (50% inhibition concentration) value was calculated about 35 μM and this concentration of baicalein was used for subsequent gene expression studies. These findings suggest that baicalein reduces the viability of Saos-2 cells.

### 3.2. Effect of baicalein on miR-25 expression 

**Figure 2:**
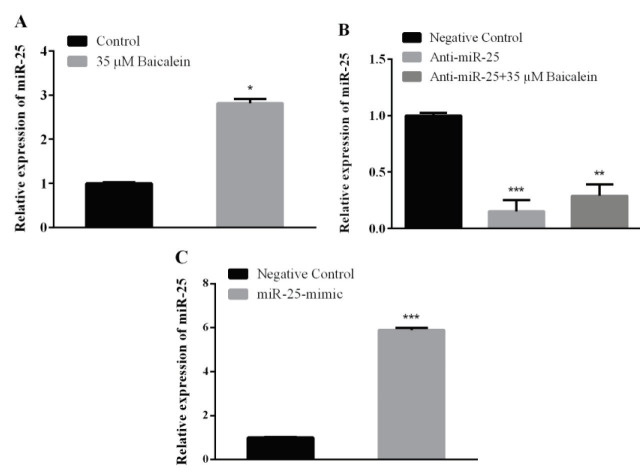
Relative expression of miR-25 mRNA in Saos-2 cells. Saos-2 cells were treated with (A) baicalein (35 μM), (B) miR-25 inhibitor (with or without baicalein) and (C) miR-25 mimic for 48 h. Then expressions were detected by qRT-PCR. RNU6B was used as an endogenous control. Values represent the mean ± standard deviation of 3 independent experiments performed in triplicate. **P < 0.01; ***P < 0.001 indicated statistical significance.

MiR-25 expression was determined by real time PCR using 35 μM baicalein for 48 h on Saos-2 cells. The expression level of miR-25 was compared with the control group (without baicalein). It was observed that baicalein statistically increased miR-25 expression (Figure 2A).

### 3.3. Effect of anti-miR-25 transfection on miR-25 expression

In order to determine the anti-miR-25 concentration to be applied to suppress miR-25 expression in Saos-2 cells, different concentrations were administered at 48 h. cDNA was synthesized from the obtained samples and miR-25 expression was evaluated. A statistically significant decrease of miR-25 expression was observed at 30 nM anti-miR-25 concentration. To evaluate baicalein’s effect we compared miR-25 expressions on anti-miR-25 transfected group and anti-miR-25 transfection + baicalein treated group against to anti-miR-25 negative control group. When we compared miR-25 expressions, we found that anti-miR-25 transfected cells were not so much affected by baicalein treatment. MiR-25 expression wasdown regulated in both groups (Figure 2B).

### 3.4. Effect of MiR-25 mimic transfection on miR-25 expression

Based on the doses recommended by the manufacturer, it was decided that the 5 nM miR-25 mimic dose was sufficient to be used to increase the miR-25 level in the cell. After miR-25 mimic transfection to the Saos-2 cells, miR-25 expression was determined by qRT-PCR. It has been observed that 5 nM miR-25 mimic was increased the miR-25 expression (Figure 2C).

### 3.5. qRT-PCR results

**Figure 3:**
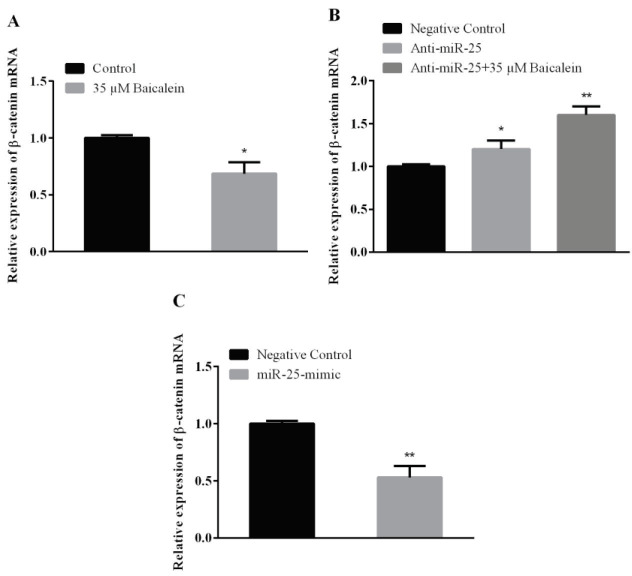
β-catenin mRNA in Saos-2 cells. Saos-2 cells were treated with (A) baicalein (35 μM), (B) miR-25 inhibitor (with or without baicalein) and (C) miR-25 mimic for 48 h. Then expressions were detected by qRT-PCR. GAPDH was used as an endogenous control. Values represent the mean ± standard deviation of 3 independent experiments performed in triplicate. *P < 0.05; **P < 0.01; ***P < 0.001 indicated statistical significance.

To determine the expression levels of Wnt/β-catenin pathway-related genes (β-catenin and Axin2), Saos-2 cells were treated with baicalein, anti-miR-25, miR-25 mimic, and their controls. When baicalein treatment was compared with its control in Saos-2 cells, it was observed that the β-catenin expression was downregulated (Figure 3A). Beta-catenin expression in anti-miR-25 and anti-miR-25 + 35 μMbaicalein groups were compared with the anti-miR-25 negative control group (Figure 3B). β-catenin expression was increased in anti-miR-25 transfected cells (with or without baicalein). When gene expression results obtained after 5 nM miR-25-mimic transfection in Saos-2 cells were compared with the miR-25-mimic negative control group, it was observed that there was a significant decrease in the expression of β-catenin mRNA (Figure 3C). 

**Figure 4:**
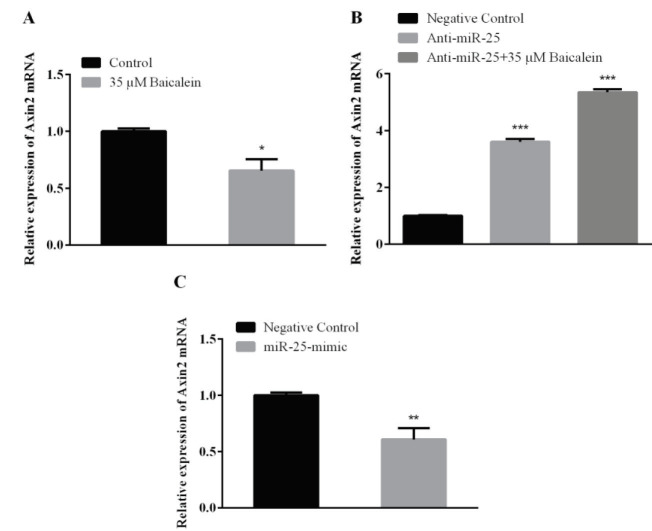
Axin2 mRNA in Saos-2 cells. Saos-2 cells were treated with (A) baicalein (35 μM), (B) miR-25 inhibitor (with or without baicalein) and (C) miR-25 mimic for 48 h. Then expressions were detected by qRT-PCR. GAPDH was used as an endogenous control. Values represent the mean ± standard deviation of 3 independent experiments performed in triplicate. *P < 0.05; **P < 0.01; ***P < 0.001 indicated statistical significance.

As a result of baicalein treatment to Saos-2 cells, Axin2 mRNA expression was evaluated. When this was compared with the control group, Axin2 expression showed a decrease (Figure 4A). Anti-miR-25 and anti-miR-25 with 35 μM baicalein treatments to Saos-2 cells were performed to determine the Axin2 expression change and the results were compared with the anti-miR-25 negative control group. A statistically significant increase in the Axin2 expression was observed in the group treated with anti-miR-25 alone and with baicalein concentration (35 μM) (Figure 4B).

Saos-2 cells were transfected with 5 nM miR-25-mimic and Axin2 gene expression was detected. The results were compared with the miR-25-mimic negative control group. The Axin2 expression was down-regulated when compared to the microRNA-25-mimic negative control group (Figure 4C). 

### 3.6. Western blot assay results

**Figure 5:**
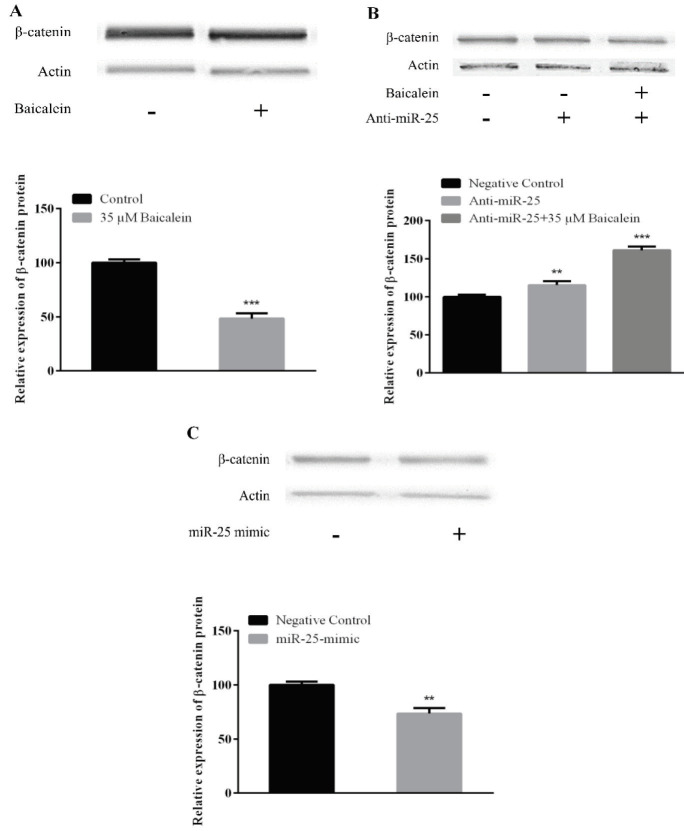
The protein levels of β-catenin. Saos-2 cells treated with (A) baicalein (35 μM), (B) anti-miR-25 (with or without baicalein) and (C) miR-25 mimic for 48 h. Then they were subjected to Western blotting to analyze the protein levels. Actin was used as an endogenous control. **P < 0.01; ***P < 0.001 indicated statistical significance.

The change in expression of β-catenin, Axin2, and GSK-3β protein was determined. When β-catenin protein expression was assessed after administration of 35 μM baicalein compared with the control group, it was found to be effective in decreasing β-catenin expression (Figure 5A, P < 0.05). β-catenin protein expression changes after anti-miR-25 transfection were determined and changes were assessed by comparing with the anti-miR-25 negative control group. β-catenin expression was increased in the groups in which anti-miR-25 was administered alone and in combination with baicalein (Figure 5B). When β-catenin protein expression results obtained after 5 nM miR-25-mimic transfection was compared with the miR-25 mimic negative control group, β-catenin protein expression was decreased (Figure 5C).

**Figure 6:**
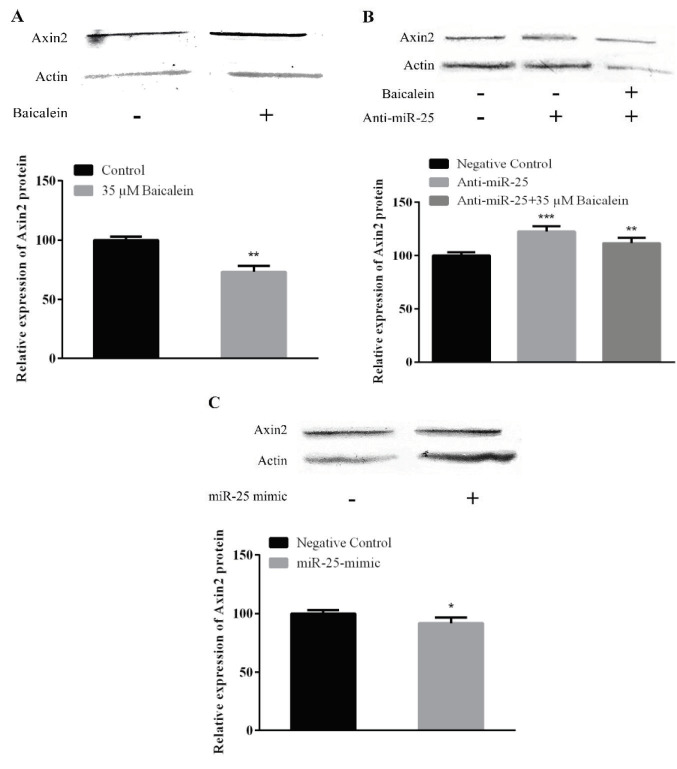
The protein levels of Axin2. Saos-2 cells treated with (A) baicalein (35 μM), (B) anti-miR-25 (with or without baicalein) and (C) miR-25 mimic for 48 h. Then they were subjected to Western blotting to analyze the protein levels. Actin was used as an endogenous control. **P < 0.01; ***P < 0.001 indicated statistical significance.

After the treatment of baicalein (35 μM), it was found that the concentration of baicalein used in comparison with the control group was effective in decreasing the Axin2 expression (Figure 6A; P < 0.05). The changes in Axin2 protein expression after anti-miR-25 transfection are shown in Figure 6B. The results were compared with anti-miR-25 negative control group to evaluate expression change. According to the results, there was an increase in the expression of Axin2 in anti-miR-25 treated group with or without baicalein (P < 0.05).Protein expression results obtained after 5 nM miR-25-mimic transfection in Saos-2 cells Axin-2 expression were statistically reduced at 48th h compared to the miR-25-mimic negative control group (Figure 6C).

**Figure 7:**
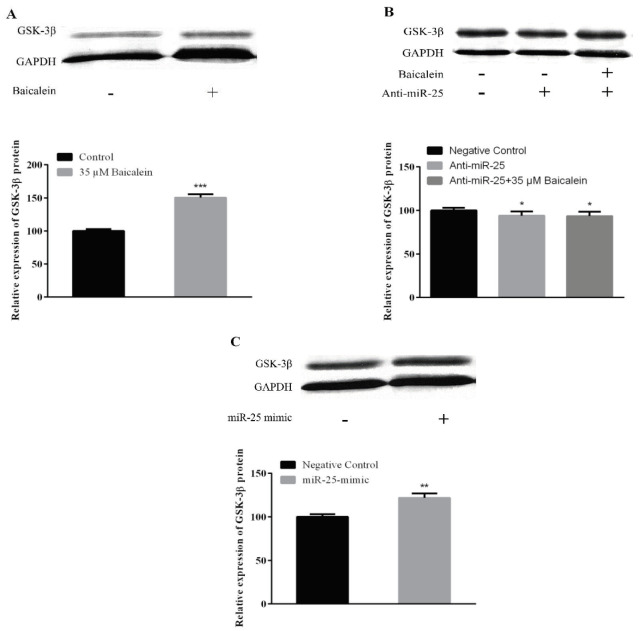
The protein levels of GSK-3β. Saos-2 cells treated with (A) baicalein (35 μM), (B) anti-miR-25 (with or without baicalein) and (C) miR-25 mimic for 48 h. Then they were subjected to Western blotting to analyze the protein levels. GAPDH was used as an endogenous control. **P < 0.01; ***P < 0.001 indicated statistical significance.

GSK-3β expression was increased compared to the control group according to the data obtained from protein expression studies to determine the effect of 35 μM baicalein on the expression of the GSK-3β protein in Saos-2 cells (Figure 7A; *P < 0.05). Changes in the expression of GSK-3β protein after anti-miR-25 transfection were determined. Expression change was assessed by comparison with the anti-miR-25 negative control group. In the groups that were treated with anti-miR-25 with or without baicalein, reduction in GSK-3β expression was reduced (Figure 7B). GSK-3β protein expression results obtained after 5 nM miR-25 mimic transfection in Saos-2 cells were compared with the mimic negative control group. GSK-3β protein expression statistically increased in this group (**P < 0.01; Figure 7C).

When the data obtained in the study are evaluated together, qRT-PCR and western blot results are supportive of each other. The change of expression in the Wnt/β-catenin pathway-related genes showed similar results in both qRT-PCR and western blot assays.

## 4. Discussion

New approaches to disease treatment and alternative practices are promising for treatment success baicalein has antitumor effects against various cancers, apoptosis-inducing and antiproliferative properties in different cell types [28,29]. Baicalein-mediated inhibition of proliferation in osteosarcoma has been reported, but the molecular mechanism of baicalein in OS is still not understood. Wnt/β-catenin signal becomes overactivated in OS and promotes tumor progression [30] and baicalein suppresses the viability of osteosarcoma cells, such as MG-63, via the Wnt signaling pathway [22]. In a study with human HEK293 cells, miRNA regulating the canonical Wnt pathway was identified, including miR-25, which inhibits β-catenin by binding the coding sequence. [25]. Our previous data showed that baicalein affects miR-25 expression on different cell lines. However, it is not certain whether the antitumor effect of baicalein is related to the modulation of miR-25 expression through the Wnt-β-catenin pathway on Saos-2.

In this study, baicalein inhibited cell proliferation in Saos-2 cells (Figure 1). The antiproliferative and apoptotic effect of baicalein has been shown in many studies. It has also been demonstrated that baicalein inhibits the growth of T24 cells by blocking the cell cycle in the G1/S phase [7]. In a recent study, baicalein has been shown to inhibit the proliferation of MG-63 osteosarcoma cells and induce apoptosis through the expression of ROS-induced BNIP3 [5]. These results indicate that the molecular targets of baicalein in various cancers are different [28,29].

In another part of our study, the effect of baicalein on the expression of miR-25 in Saos-2 cells and also the effect of altering miR-25 expression on the expression of genes in the Wnt/β-catenin pathway were investigated. According to our results, baicalein induces miR-25 expression on Saos-2 cells (Figure 2A). When we look at the expression of genes in the Wnt/β-catenin pathway after baicalein treatment; the expression of β-catenin and Axin2 was downregulated in both protein and mRNA levels (Figures 3A, 4A, 5A, and 6A). Β-catenin is an important molecule in the Wnt/β-catenin signaling pathway and is important for the activation status of the Wnt/β-catenin pathway in the transition between the cytoplasm and the nucleus in osteosarcoma cells [31]. Translocation of β-catenin from cytoplasm to nucleus regulates transcription of many genes involved in cellular processes [32]. Expressions of genes such as β-catenin, GSK-3β, and Axin2 are targeted in Wnt pathway studies. The change in gene expression indicates that the pathway is active or inactive. Down-regulation of β-catenin inactivates Wnt/β-catenin pathway. Increased β-catenin levels can stimulate transcriptional activation of proteins such as c-Myc, which controls the passage of G1 to S phase in the cell cycle, leading to DNA replication and mitosis [33]. The increased expression of β-catenin is strongly associated with poor prognosis in breast cancer patients. This accumulation can be due to many factors such as β-catenin mutations, deficiencies in β-catenin degradation complex and overexpression of Wnt ligands [34]. Baicalein inhibits cell proliferation by targeting the c-myc gene through the Wnt signaling pathway and induce apoptosis on MG-63 osteosarcoma cell line. Baicalein also decreases the expression of β-catenin [22]. Similarly, studies with different cell lines have shown that baicalein affects the Wnt/β-catenin pathway by targeting β-catenin [3]. Axin2 (Axis inhibition protein 2) is a transcriptional target of β-catenin/TCF. The Axin2 expression is thought to be a sign of the activation of the Wnt pathway [35]. In our study, the treatment of baicalein caused a decrease in the expression of Axin2 (Figures 4A and 6A). This reduction seems to be related to the decrease in the expression of β-catenin and hence the reduction of translocation in the nucleus. The translocation of β-catenin to the nucleus activates the transcription of the target Wnt pathway genes such as *c-Myc, c-jun, cyclin D1* and *Axin2 *[36]. Ma et al. [37] showed that baicalein can inhibit the expression of Wnt1 and β-catenin in breast adenocarcinoma cells and decreased both the expression of Cyclin D1 and the core Axin2 expression at the level of transcription by dose and time.

We also investigated the effect of baicalein on the expression of miR-25 in Saos-2 cells and also the effect of altering miR-25 expression on GSK-3β. We found an increase in GSK-3β protein expression by baicalein treatment (Figure 7A). GSK-3β is found in the cytoplasm as part of the destruction complex which contains Axin and adenomatous polyposis coli (APC). GSK-3β causes rapid phosphorylation of cytoplasmic β-catenin. This posttranslational phosphorylation by the degradation complex of β-catenin makes the β-catenin a target for ubiquitination and subsequent proteasomal degradation, thus preventing nuclear accumulation of β-catenin [38]. Suzuki et al. [39] reported that Wnt/β-catenin signaling pathway was activated by inactivation of GSK-3β in Saos-2 cells. It was reported that the expression of Axin protein in GSK-3β was increased after baicalein treatment in MG-63 osteosarcoma cells [22]. This data is in parallel with the results we have obtained. Our results suggest that increased GSK-3β due to baicalein treatment causes β-catenin reduction by ubiquitination and proteasomal degradation [32].

The change in the presence, absence or amounts of microRNAs in cells and tissues enables them to control gene expression in a certain cellular process. One of the best ways to understand the microRNAs is to elucidate functional targets. This is possible by analyzing the changes in target genes or pathways following gain or loss of function of the specific microRNA. After we found out the effect of baicalein on the Wnt/β-catenin pathway, we also investigated the relationship between increased miR-25 expression with baicalein treatment over this pathway. MiR-25 expression was reduced by transferring miR-25-inhibitor with baicalein (Figure 2) and was increased by transferring miR-25 mimic without baicalein to determine whether the miRNA-25 expression was effective in the Wnt/β-catenin pathway by itself (Figures 2B and 2C). Anti-miR-25 with and without baicalein treated cells both showed increases on β-catenin and Axin2 mRNA and protein expression (Figures 3B, 4B, 5B, and 6B) and decreases on GSK-3β protein expression (Figure 7B). In the case of miR-25 mimic transfection without baicalein treatment, a decrease in mRNA and protein expressions for β-catenin and Axin2 (Figures 3C, 4C, 5C, and 6C) and an increase in expression of the GSK-3β protein (Figure 7C) were observed. These results show a direct effect of miR-25 on the Wnt/β-catenin pathway. 

Studies have shown that variation in the expression of microRNAs regulates the components of the Wnt/β-catenin signaling pathway. Both miRNAs and Wnt signaling pathways interact to regulate variety of biological processes [24,38,40]. In a study of miRNAs involved in the Wnt signaling pathway, it was reported that miR-25 is involved in this pathway and inhibits the proliferation of colon cancer cells and cause the inhibition of the Wnt/β-catenin pathway [25]. Coadministration of anti-miR-25 with baicalein and mimic transfection show that miR-25 is effective in modulating β-catenin, Axin2 and GSK-3β expression in the regulation of the Wnt/β-catenin pathway. 

When all of the data obtained in the study are evaluated together, it demonstrates that baicalein may inhibit cell proliferation in Saos-2 cells. The expression of the GSK-3β protein was increased when the mRNA and protein levels of β-catenin and Axin2, which are located on the pathway of Wnt/β-catenin and give information about the activity of pathway, are decreased after administration of baicalein at the IC50 concentration. Increased expression of GSK-3β decreases the level of β-catenin in the cytoplasm and decreases the amount of β-catenin that enters the nucleus [38]. This reduction leads to a decrease in Axin2 protein level and blockage of the Wnt/β-catenin pathway. The effect of baicalein on the Wnt/β-catenin pathway seems to be related to miR-25 expression. Increased miR-25 expression by baicalein administration changes the expression of β-catenin, Axin2, and GSK-3β genes, as demonstrated by inhibitor and mimic transfections. 

In conclusion, the effect on these genes shows that miR-25 regulates the Wnt/β-catenin pathway in the Saos-2 cell line. Baicalein appears to have an anticancer effect on the cells. Baicalein targeting the Wnt/β-catenin signaling pathway in the Saos-2 suggests that it may be used as a therapeutic in osteosarcoma. Also, the effect of baicalein on the Wnt/β-catenin pathway paralleled with the miR-25 gene expression, suggests that the molecular mechanism is related to miR-25 expression. In order to support the study data, it is important to determine which Wnt/β-catenin signal pathway related genes are the direct target gene of miR-25 by different procedures. In this molecular mechanism, more detailed studies are needed to elucidate the target genes of miR-25 and the relationship between proliferation and apoptosis through the Wnt/β-catenin signaling pathway.

## Acknowledgments

This study was supported by Aydın Adnan Menderes University Scientific Research Projects (Grant Number FEF-15019). We thank TARBIYOMER and REDPROM (Aydın Adnan Menderes University) for sharing their laboratory. 

## Conflict of interest

The authors declare no conflict of interest.

This study was presented at the Taiwan-Turkey Science Summit entitled “Translation of Cells, Nanomaterials and Signaling Molecules into Regenerative Medicine” between April 1 to 3, 2018.
